# Enhanced dissolution of silver nanoparticles (10 nm) in the presence of platinum nanoparticles (3 nm) causes increased cytotoxicity: mechanistic insight *via* transmission electron microscopy and X-ray powder diffraction

**DOI:** 10.1039/d5ra02579f

**Published:** 2025-06-26

**Authors:** O. Prymak, M. Breisch, K. Loza, M. Heggen, M. Köller, C. Sengstock, M. Epple

**Affiliations:** a Inorganic Chemistry, Center for Nanointegration Duisburg-Essen (CENIDE), University of Duisburg-Essen Universitaetsstr. 5-7 45117 Essen Germany matthias.epple@uni-due.de; b BG University Hospital Bergmannsheil, Surgical Research, Ruhr University Bochum Bochum Germany; c Ernst Ruska-Centre for Microscopy and Spectroscopy with Electrons, Forschungszentrum Jülich GmbH 52425 Jülich Germany; d ISAS Leibniz-Institut für Analytische Wissenschaften – ISAS – e.V. Bunsen-Kirchhoff-Str.11 44139 Dortmund Germany

## Abstract

The dissolution of silver nanoparticles (10 nm) is strongly enhanced by the presence of platinum nanoparticles (3 nm) in chloride-containing aqueous dispersion. This was shown using X-ray powder diffraction and transmission electron microscopy. Direct contact between the two metals is necessary as shown by transmission electron microscopy. Complete dissolution of silver nanoparticles occurs within about one hour in the presence of potassium chloride as the electrolyte but not in pure water. Thus, the dissolution of silver nanoparticles in aqueous dispersion requires the presence of platinum nanoparticles for polarization as well as the presence of electrolyte ions for charge balancing. After dissolution, the silver ions precipitate as silver chloride. A minor part of silver is taken up by platinum to form a solid solution (alloy). The enhanced release of silver ions in the dispersion of nanoparticles of both metals leads to a decreased viability of human mesenchymal stem cells (hMSC), caused by the cytotoxic effect of silver ions.

## Abstract 2

The dissolution of silver nanoparticles (10 nm) is strongly enhanced by the presence of platinum nanoparticles (3 nm) in chloride-containing aqueous dispersion.

## Graphical abstract

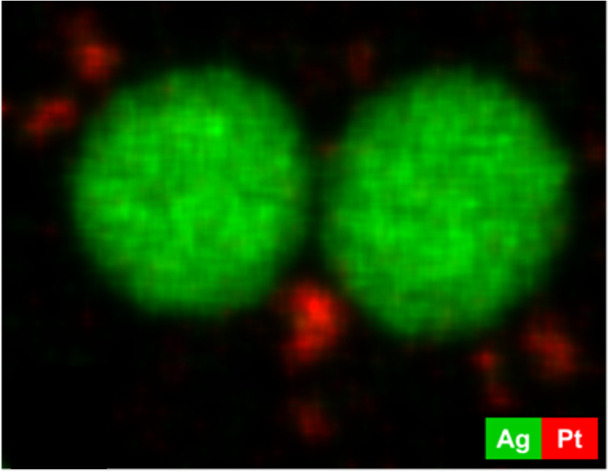

## Introduction

Silver nanoparticles are used in a wide range of applications, including biomedicine, due to their special physical and chemical properties.^1–7^ Their antimicrobial properties, which are caused by the release of silver ions,^8–11^ make them particularly attractive for the surface coating for implants, in wound dressings and other biomedical products. In general, the ion release can be adjusted by the size of nanoparticles,^12,13^ the presence of oxygen,^14–21^ and the presence of electrochemically more noble metals like platinum.^22–24^ Silver nanoparticles show a slow dissolution in aqueous media under release of silver ions which have a distinct antibacterial and cytotoxic effect.^15,20,25–30^

One application of silver is the creation of antimicrobial surfaces, because an increase in the silver ion concentration from an implant surface will induce local antibacterial activity. An increase in the ion release from silver-containing coatings is necessary in some clinical applications where an initial burst of silver ions helps to prevent bacterial adhesion.^31,32^ We have previously shown that a pure silver coating does not release sufficient silver ions to keep an implant surface sterile within an infected tissue-like environment due to the high local protein concentration combined with a slow diffusion of silver ions in the biological environment.^33^ However, an electrochemically enhanced silver dissolution leading to increased silver ion release occurs from thin silver films that were sputtered onto platinum deposits.^24,33^ We have also shown that silver nanoparticles experience enhanced silver ion release in the presence of platinum nanoparticles in dispersion.^22^ This effect is probably due to polarization of silver by platinum (sacrificial anode effect). However, the microscopic mechanism of this dissolution remains unclear.

Here we present a detailed electron microscopic and crystallographic study that shows how the dissolution of silver nanoparticles is strongly enhanced by the local presence of platinum nanoparticles.

## Experimental part

### Chemicals and reagents

For the synthesis of silver nanoparticles, we used silver nitrate (Carl Roth, 99%), trisodium citrate dihydrate (AppliChem GmbH, p.a.), and sodium borohydride (Fluka, 96%). The platinum nanoparticles were synthesized with hexachloridoplatinum acid (H_2_PtCl_6_, Sigma-Aldrich) and d-glucose (Sigma Aldrich, ≥99.5%). The nanoparticles were colloidally stabilized with poly(*N*-vinylpyrrolidone) (PVP, Povidon 30, Fluka, *M* = 40 000 g mol^−1^). Ultrapure water with a specific resistivity of 18.2 MΩ was prepared with a Purelab ultra instrument (ELGA) and used for all experiments unless noted otherwise. All glassware was cleaned with boiling *aqua regia* and rinsed thoroughly with water before all experiments.

### Synthesis of silver nanoparticles (10 nm)

The synthesis was carried out as reported earlier.^16^ Briefly, 18 mg (0.06 mmol) silver nitrate and 22 mg (0.1 mmol) trisodium citrate dihydrate were added to 330 mL degassed ice-cold water. Under vigorous stirring, 5 mL of a freshly prepared 20 mM sodium borohydride solution was rapidly added to the mixture. After one minute, 300 mg PVP dissolved in 10 mL were rapidly injected. The reaction mixture was stirred for 3 h at 0 °C during cooling with an ice bath. Then, the reaction volume was reduced to 50 mL in a rotary evaporator (60 °C, 95 mbar). The particles were isolated and purified by threefold centrifugation at 20 000 rpm (29 400*g*). The particles were well dispersible in water and stored in degassed water under argon atmosphere at 8 °C to prevent premature dissolution by oxidation from air or dissolved oxygen^16^ until further use.

### Synthesis of platinum nanoparticles (3 nm)

The synthesis was carried out as reported earlier.^34^ Briefly, 600 mg (3 mmol) d-glucose and 50 mg PVP were dissolved in 50 mL water. The reaction mixture was heated under stirring to 100 °C under reflux. Then 5 mL of an aqueous solution of hexachloridoplatinum acid (25 μmol) was rapidly added, and the reaction mixture was boiled for 4 h under reflux. The synthesis byproducts were removed by spin filtration (2500*g*) (Millipore; molecular weight cut-off 3 kDa). Finally, the particles were isolated and purified by centrifugation at 30 000 rpm (66 000*g*) for 30 min. The particles were well dispersible in water and stored in degassed water under argon atmosphere until further use.

### Analytical methods

Silver and platinum concentrations in the nanoparticle dispersions were determined by atomic absorption spectroscopy (AAS) with a ThermoElectron M-Series spectrometer (graphite tube furnace operated according to DIN EN ISO/IEC 17025:2005) after dissolving the nanoparticles in concentrated nitric acid (Ag) and *aqua regia* (Pt), respectively. UV-vis spectroscopy was performed on water-dispersed nanoparticles with a Varian Cary 300 instrument from 200 to 800 nm after background solvent correction in Suprasil® quartz glass cuvettes with a sample volume of 3 mL.

### Electron microscopy

High-resolution imaging was performed with an aberration-corrected FEI Titan transmission electron microscope equipped with a Cs-probe corrector (CEOS Company), operated at 300 kV.^35^ Scanning transmission electron microscopy (STEM) was performed with a FEI Titan microscope, equipped with Cs-probe corrector (CEOS Company) and a high-angle annular dark field (HAADF) detector, operated at 200 kV.^36^*Z*-Contrast conditions were achieved with a probe semi-angle of 25 mrad and an inner collection angle of the detector of 70 mrad. Elemental mapping by energy-dispersive X-ray spectroscopy (EDX) was conducted with a probe-corrected FEI Titan 80-200 “ChemiSTEM” electron microscope equipped with four symmetrical SDD detectors.^36^

### X-ray powder diffraction (XRD)

X-ray powder diffraction was carried out with a Bruker D8 Advance diffractometer in Bragg–Brentano geometry with Cu Kα radiation (*λ* = 1.5418 Å; *U* = 40 kV, *I* = 40 mA). All samples were investigated from 20 to 90° 2*θ* with a step size of 0.02° and a counting time of 8 s, resulting in a total measurement time of 8.4 h. 100 μL of concentrated aqueous colloidal dispersions of Ag and Pt nanoparticles with *c*(Ag) = *c*(Pt) = 0.58 mg mL^−1^ and their 1 : 1 mixture (v : v), respectively, were placed on a silicon single crystal sample holder and gently dried with a flow of warm air. This led to a homogeneous dense circle of nanoparticles with a diameter of about 16 mm, *i.e.* at least the size of the X-ray beam spot. To study the dissolution behavior in the Ag/Pt nanoparticle mixture in contact with electrolytes, 200 μL of 3 M KCl solution was dripped onto the dried nanoparticles on the silicon sample holder and kept there for 30 min. Then, water was gently evaporated by drying with warm air, and X-ray powder diffraction was performed.

Qualitative phase analysis was performed with the program Diffrac.Suite EVA V1.2 from Bruker with the ICDD database patterns of Ag (#04-0783), Pt (#04-0802), KCl (#73-0380), and AgCl (#31-1238). For the determination of the unit cell parameters and the average crystallite size (CS), Rietveld refinement was carried out with the Bruker software TOPAS 5.0, taking into account the instrumental peak broadening as determined previously with an LaB_6_ standard (microcrystalline powder from NIST; SRM 660b).^37^ The crystallite size CS was calculated from the diffraction peak broadening with the Scherrer and Stokes–Wilson equation^38^ integrated in the Rietveld refinement package TOPAS:
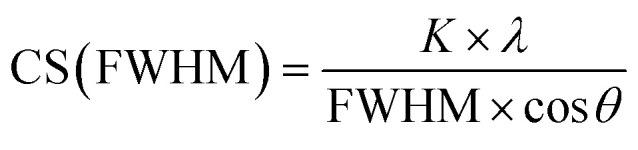
where *K* is a constant set to 0.89 (assuming a spherical particle shape), *λ* is the wavelength of the X-radiation, FWHM is the full width at half-maximum of the diffraction peaks in radians (after considering the instrumental peak broadening), and *θ* is the diffraction angle.

### Cell culture

Human mesenchymal stem cells (hMSC; 5th to 10th passage; Lonza, Basel, Switzerland) were cultivated in cell culture medium RPMI1640 (GIBCO, Invitrogen, Karlsruhe, Germany) containing 10% fetal calf serum (FCS; GIBCO) and 0.3 g L^−1^l-glutamine (RPMI/FCS) in 75 cm^2^ culture flasks (BD Falcon, Becton Dickinson GmbH, Heidelberg, Germany). The cells were grown in humidified 5% CO_2_ atmosphere at 37 °C (standard cell culture conditions) and sub-cultivated every 7 to 14 days, depending on the cell proliferation.

Adherent subconfluently growing hMSC were detached from the culture flasks by washing with phosphate-buffered saline solution (PBS; GIBCO), followed by adding 0.2 mL cm^−2^ of 0.05% trypsine/0.02% ethylenediaminetetraacetic acid (EDTA, Sigma-Aldrich, Taufkirchen, Germany) solution for 5 min at 37 °C. The detached cells were harvested, washed twice with RPMI/FCS and seeded at a density of 1.5 × 10^4^ cells per well in 24-well cell culture plates (BD Falcon).

### Cell viability

Adherent hMSC were exposed to dispersions of pure silver nanoparticles, pure platinum nanoparticles, or a physical 1 : 1 (v : v) mixture of silver and platinum nanoparticles for 2 h, 4 h, 16 h, and 7 days, respectively, in RPMI/FCS under standard cell culture conditions. Silver and platinum nanoparticle dispersions were mixed at a 1 : 1 volume ratio immediately before each experiment to avoid premature silver oxidation by air.

After nanoparticle exposure, the cell viability was assessed by a live-dead assay. Living cells were visualized by staining with 1 μM calcein–acetoxymethylester (calcein–AM; Calbiochem, Schwalbach, Germany; 30 min, 37 °C) and dead cells by staining with 50 μg mL^−1^ propidium iodide (PI; Sigma-Aldrich; 10 min, room temperature), followed by fluorescence microscopy (Olympus MVX10, Olympus, Hamburg, Germany). Quantification of the cell viability was performed by optical phase analysis (cellSens Dimensions, Olympus) by calculating the calcein-fluorescent area. The data for hMSC treated with nanoparticles are given as percentage of the non-treated hMSC control which was set as 100%.

## Results and discussion

**Fig. 1:**
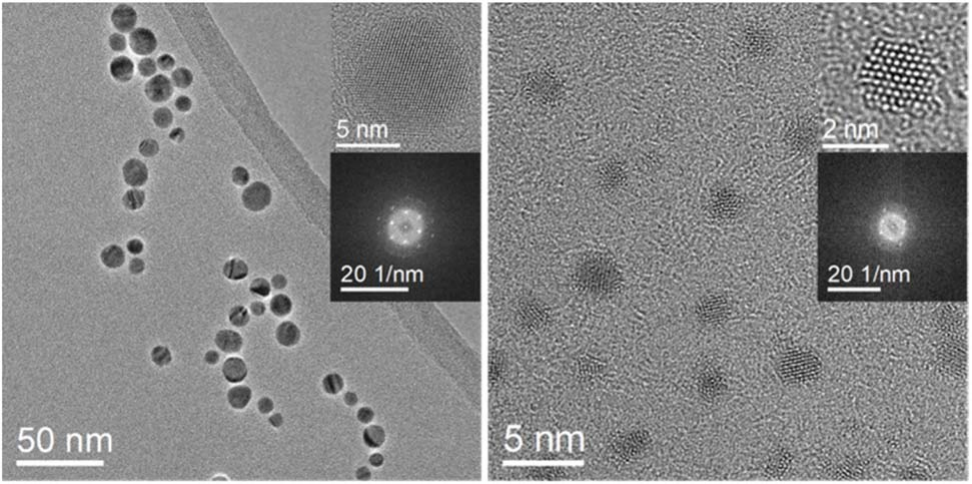
Transmission electron micrographs and corresponding FFT insets of silver (left) and platinum (right) nanoparticles.

The enhanced dissolution of silver nanoparticles in the presence of platinum nanoparticles was studied on the microscopic scale. Of course, a prior in-depth characterization of the nanoparticles used is crucial to understand their physical and chemical properties. The PVP-stabilized platinum and silver nanoparticles were well dispersible in water and monodisperse. The UV-vis absorption spectrum of the silver nanoparticles showed the typical surface plasmon resonance band at 390 nm (see ref. 16 for the spectrum) whereas the spectrum of the platinum nanoparticles showed no absorption band at all (see ref. 39 for the spectrum). Transmission electron microscopy confirmed the uniformity and the high internal crystallinity of both types of nanoparticles with a particle size of about 10 nm for silver and 3 nm for platinum nanoparticles. Many nanoparticles were crystallographically twinned as found earlier for such particles (Fig. 1).^34^

**Fig. 2:**
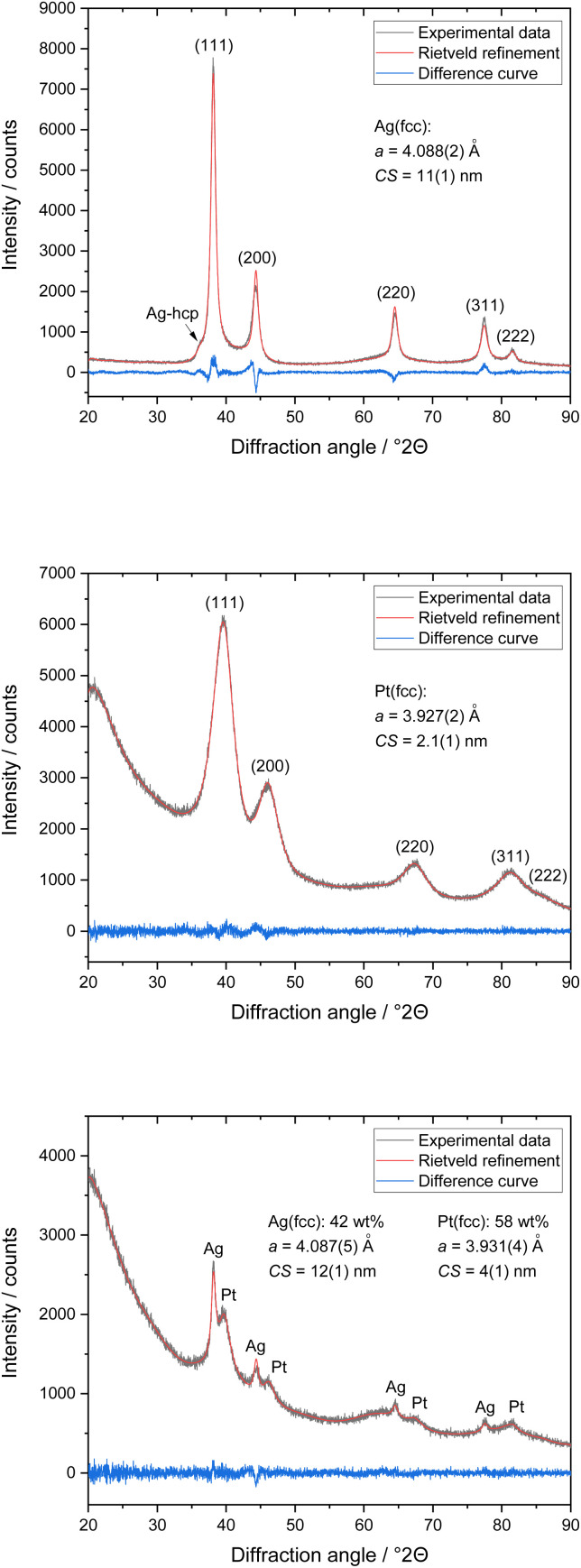
X-ray powder diffraction data and Rietveld refinement of silver nanoparticles (top) and platinum nanoparticles (center). Miller indices of silver and platinum as well as a small peak shoulder of an Ag-hcp phase (≈1 wt%) are shown. Bottom: X-ray powder diffractogram with Rietveld refinement of a physical mixture of silver and platinum nanoparticles showed both kinds of nanoparticles. The mass ratio in the mixture as determined by Rietveld refinement was Ag : Pt = 42 : 58 wt%, in good agreement with the expectations (50 : 50 wt%).

X-ray powder diffraction (Fig. 2) showed that silver and platinum nanoparticles were crystallographically pure without other phases or contaminations. The fcc lattice parameters *a*(Ag) = 4.088 Å and *a*(Pt) = 3.927 Å by Rietveld refinement agreed well with the ICDD database. The silver nanoparticles occurred in the fcc structure as expected, but a small amount of hcp silver metal was found (which is not unusual for silver nanoparticles).^40^ A comparison of the crystallite size (CS) with the particle size *D* from TEM showed that the silver nanoparticles (CS = 11 nm) and the platinum nanoparticles (CS = 2.1 nm) consisted of crystalline domains with a size close to the diameter of the particles.

Dispersions of silver and platinum nanoparticles were mixed in water in the volume ratio 1 : 1 with the final concentrations of *c*(Ag) = *c*(Pt) = 0.58 mg mL^−1^. 100 μL of the Ag/Pt mixture was dripped onto a silicon single crystal sample holder and dried with warm air. Quantitative X-ray powder diffraction (Rietveld refinement) of the dried nanoparticles confirmed the unchanged physical mixture of silver and platinum nanoparticles with a phase ratio Ag : Pt = 42 : 58 wt% (Fig. 2). The difference to the expected value (50 : 50 wt%) is attributed to the fact that a small amount of hcp silver was not taken into account.

**Fig. 3:**
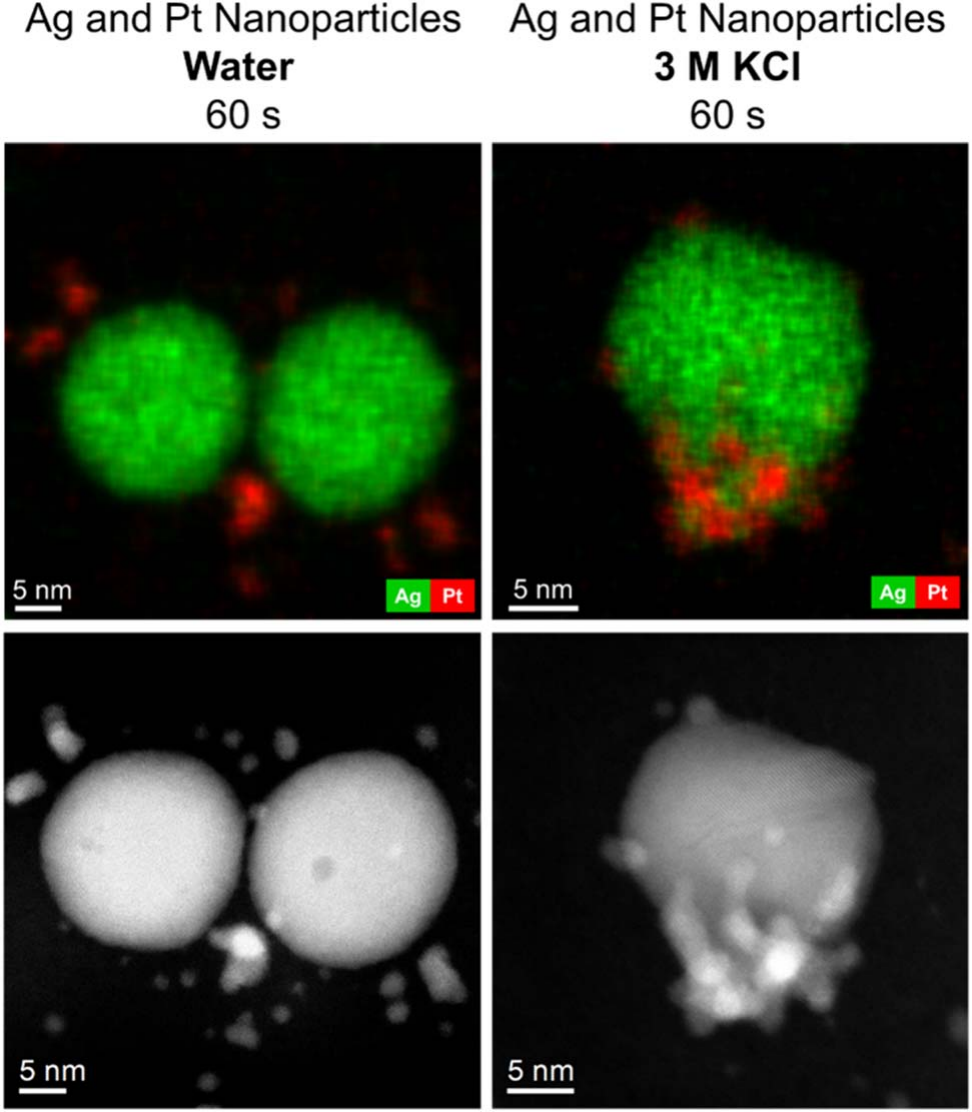
Energy-dispersive X-ray maps (top) and corresponding HAADF-STEM micrographs of silver and platinum nanoparticles (bottom) after immersion in water (left) or 3 M KCl solution (right) for 60 s.

The enhanced dissolution of silver nanoparticles in the presence of platinum nanoparticles was investigated by TEM and energy-dispersive X-ray spectroscopy (EDX). The physical mixture of dispersed silver and platinum nanoparticles (1 : 1) was drop-cast onto a carbon-coated copper grid. After drying at room temperature and ambient pressure, the grids with the dried nanoparticles were quickly immersed into either water or 3 M aqueous potassium chloride solution as model electrolyte in electrochemistry. After immersion for 60 s, the grids were gently rinsed with distilled water and dried under ambient conditions. Fig. 3 shows transmission electron micrographs and corresponding EDX maps of the physical mixture of silver and platinum nanoparticles after immersion.

**Fig. 4:**
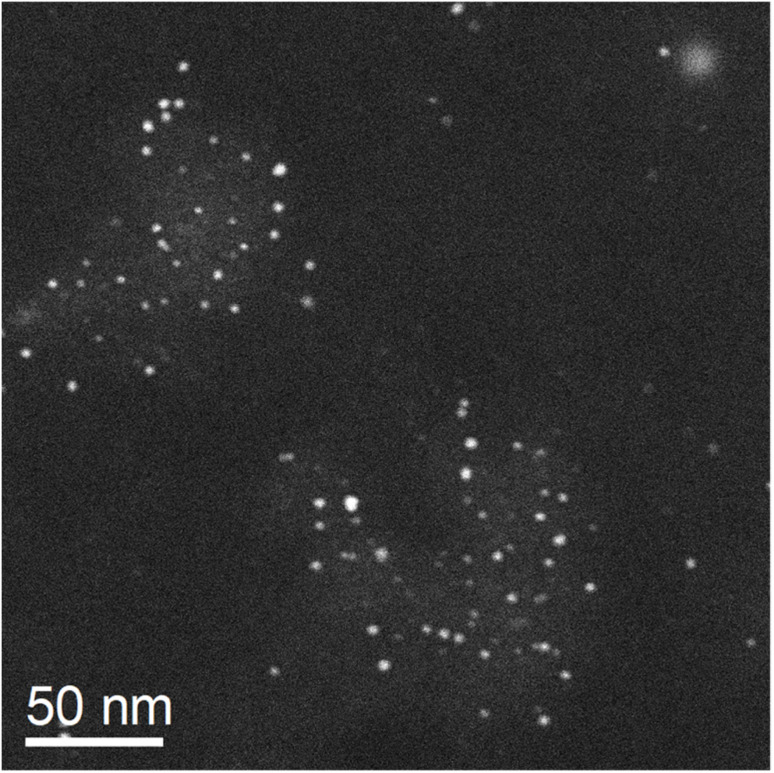
HAADF-STEM image of the nanoparticle sample after 1 h incubation of a physical Ag/Pt mixture in 3 M KCl. Only particles in the ∼3 nm size range were observed; the ∼10 nm silver nanoparticles that had been present in the original dispersion were no longer detectable.

After immersion in water, the nanoparticles showed no visual change by TEM. In contrast, the dissolution of silver was rapid after immersion into 3 M KCl solution already after 60 s. After incubation for one hour, the silver nanoparticles (10 nm) had completely dissolved, and only smaller nanoparticles (probably platinum) remained (Fig. 4).

**Fig. 5:**
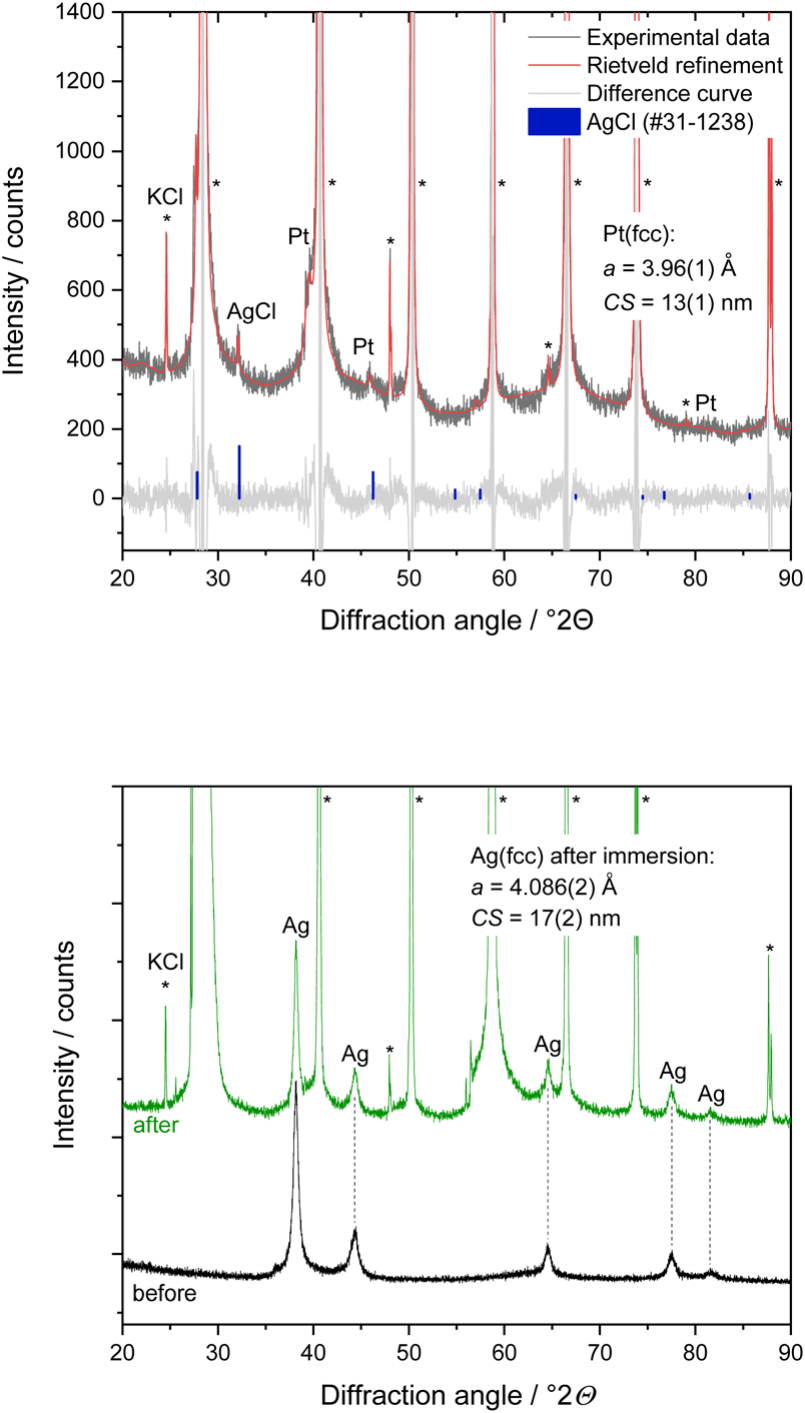
Top: X-ray powder diffractogram with Rietveld refinement of a physical mixture of silver and platinum nanoparticles after contact with 200 μL 3 M KCl solution for 30 min. Diffraction peaks of silver were absent, indicating a rapid dissolution of silver nanoparticles in 3 M KCl solution in the presence of platinum nanoparticles. Bottom: X-ray powder diffractograms of silver nanoparticles before and after treatment with 200 μL 3 M KCl solution for 10 min. No AgCl was found, indicating that silver nanoparticles had not dissolved in the absence of platinum. The diffraction peaks of the co-crystallized by-product KCl (*, split peaks due to Cu Kα1,2 radiation) are labelled.

X-ray powder diffraction performed during nanoparticle dissolution on the XRD sample holder gave additional insight. 200 μL of 3 M KCl solution was put onto the dried nanoparticles on the XRD sample holder. The dispersion was analysed again by powder diffraction after 30 min contact time, followed by drying with warm air for about 10 min (Fig. 5). X-ray powder diffraction of a physical mixture of silver and platinum nanoparticles after incubation with KCl solution and subsequent drying showed the microcrystalline phase KCl (>99.6 wt%). Furthermore, nanocrystalline AgCl (CS = 50 nm) and platinum (CS = 13 nm) in the mass ratio of about 1 to 3 were found. The diffraction peaks of silver were not detectable anymore, confirming the complete dissolution of silver nanoparticles after contact with KCl solution in the presence of platinum nanoparticles. The silver ions released from the silver nanoparticles reacted with chloride ions from the immersion medium under formation of AgCl as expected due to the solubility product.^41^ The slight increase of the lattice parameter of platinum by about 0.9% suggests a partial alloying of platinum with silver, forming a solid solution. In contrast, the Ag/Pt mixture in contact with pure water did not show a dissolution, underscoring the necessity of an electrolyte to facilitate dissolution.

In another control experiment, dried silver nanoparticles on an XRD sample holder were brought into contact with 200 μL 3 M KCl solution for 10 min. In the absence of platinum nanoparticles, the silver nanoparticles were not affected by the presence of the KCl solution, and no AgCl salt was formed (Fig. 5). The unchanged lattice parameter of the silver nanoparticles before and after the KCl treatment confirmed that the silver nanoparticles remained unchanged. All results from X-ray powder diffraction are summarized in Table 1.

**Table 1:** Lattice parameters (*a*) and crystallite sizes (CS) of as-prepared and of mixed silver and platinum nanoparticles after contact with water or 3 M KCl as determined by X-ray powder diffraction and Rietveld refinement. Standard deviations are given in parentheses

Sample	Silver nanoparticles		Platinum nanoparticles		Other crystalline phases	Dissolution of silver nanoparticles
	*a*/Å	CS/nm	*a*/Å	CS/nm		
As-prepared nanoparticles dispersed in H_2_O (only one metal in each dispersion)	4.088(2)	11(1)	3.927(2)	2.1(1)	—	No
Physical mixture of Ag and Pt nanoparticles, dispersed in H_2_O for 30 min	4.087(1)	12(1)	3.931(4)	4(1)	—	No
Physical mixture of Ag and Pt nanoparticles, dispersed in 3 M KCl solution for 30 min	—	—	3.962(5) (PtAg alloy)	13(3)	KCl, AgCl	>95%
Ag nanoparticles dispersed in 3 M KCl solution for 10 min	4.086(2)	17(2)	—	—	KCl	No

The microscopic process that occur during the dissolution of the silver nanoparticles are complex. Besides the anodic oxidation of silver to silver(+I) cations, there are local equilibria, mainly the precipitation of silver chloride. Second, at high chloride concentrations, there is also the possibility of the formation of dichloridoargentate(+I) complexes, [Ag(Cl)_2_]^−^.^14,41^ These equilibria are time-dependent, chemically coupled and can also include a local precipitation, followed by a redissolution. Diffusion is also likely to play a role. Further in-depth studies would be required to elucidate the underlying reactions in detail.

To assess the biological effects of the enhanced silver ion release induced by the presence of platinum nanoparticles, cell culture studies with human mesenchymal stem cells (hMSC) were performed. An enhanced silver nanoparticle dissolution in the presence of platinum nanoparticles and electrolytes (as present in cell culture medium) results in an enhanced silver ion release. Consequently, cell-toxic effects occur faster for Ag/Pt nanoparticle mixtures than for pure Ag nanoparticles.

**Fig. 6:**
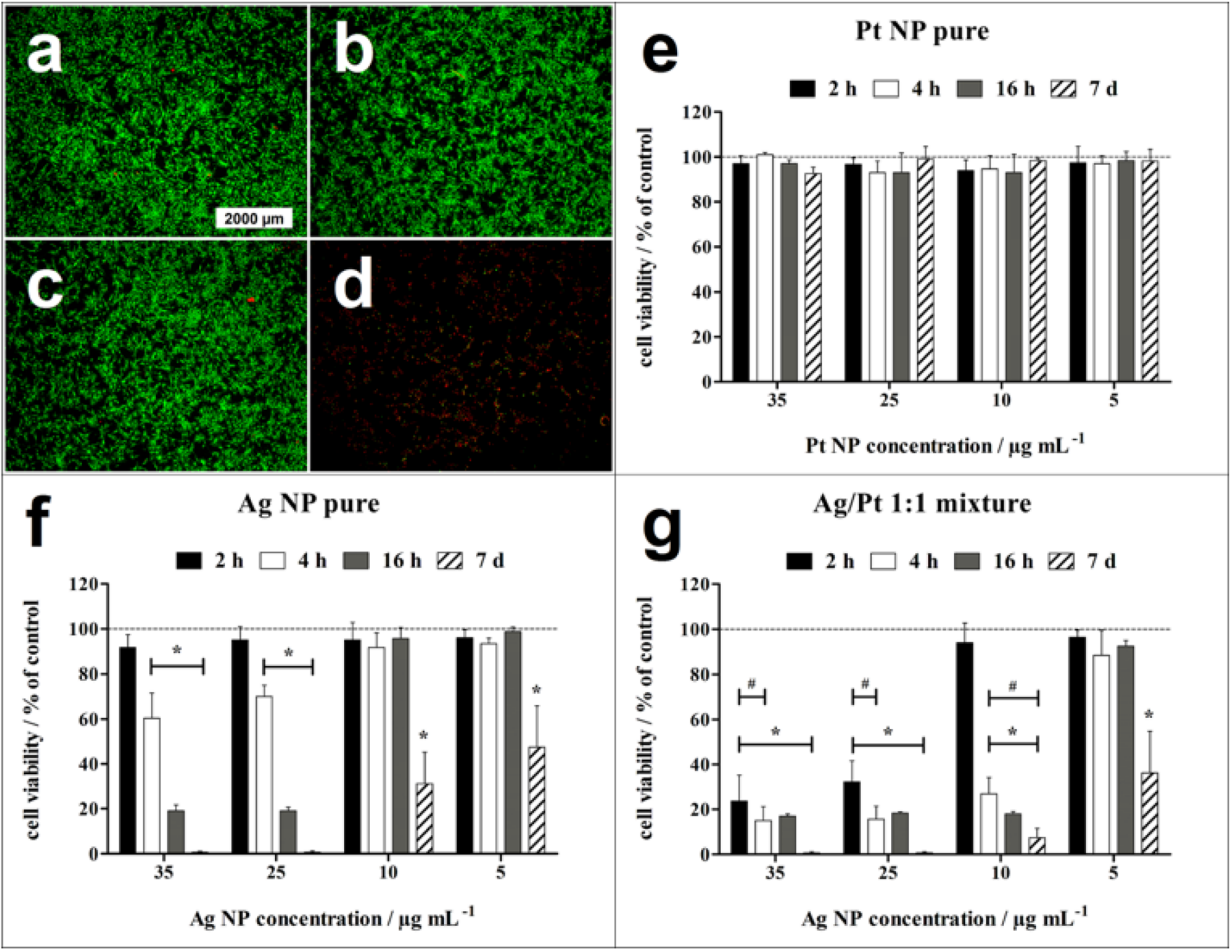
Time- and concentration-dependent effect of nanoparticles on the viability of hMSC. (a)–(d) Representative fluorescence micrographs of the live-dead staining of hMSC with calcein–AM (green fluorescence) and propidium iodide (red fluorescence) after 24 h of incubation in RMPI/FCS in the absence of nanoparticles (a, control), in the presence of pure platinum nanoparticles (b, 10 μg mL^−1^), of pure silver nanoparticles (c, 10 μg mL^−1^), and of a physical Ag/Pt 1 : 1 nanoparticle mixture (d, 10 μg mL^−1^ of each metal). (e)–(g) Quantification of hMSC viability by phase analysis of the calcein-fluorescent area after different incubation times in the presence of pure platinum nanoparticles (e), of pure silver nanoparticles (f), and of the physical Ag/Pt 1 : 1 nanoparticle mixture (g). Data are expressed as mean ± SD (*n* = 3) and given as percentage of hMSC control (100%, no nanoparticle exposure). * indicates significant differences (*p ≤ 0.05) compared to the hMSC control. # indicates significant differences (*p ≤ 0.05) compared to pure silver nanoparticles.

Fluorescence micrographs of untreated hMSC as well as of hMSC exposed to 10 μg mL^−1^ of either platinum nanoparticles or silver nanoparticles exhibited mainly the green fluorescence associated with living cells after 24 h of incubation. In contrast, the physical Ag/Pt 1 : 1 mixture containing 10 μg mL^−1^ of Ag nanoparticles led to complete loss of cell viability, as indicated by red fluorescence of dead cells (Fig. 6).

Quantitative analysis of hMSC viability after 2 h, 4 h, 16 h, and 7 days exposure to different nanoparticle concentrations showed no significant cell toxicity for pure platinum nanoparticles even after 7 days of exposure up to a nanoparticle concentration of 35 μg mL^−1^. For pure silver nanoparticles, cell-toxic effects depended on the incubation time and the nanoparticle concentration. Significant cell-toxic effects were observed after 4 h of incubation at silver nanoparticle concentrations of 25 to 35 μg mL^−1^, whereas at 5 to 10 μg mL^−1^, a significant cell toxicity was detected only after a prolonged incubation period of 7 days. This indicates a slow Ag^+^ release *via* oxidation of silver nanoparticles under these conditions until a cytotoxic concentration is reached in accordance with earlier results. The cytotoxic species are the released silver ions.^27,29,42^ In contrast to pure silver nanoparticles, the physical Ag/Pt 1 : 1 mixture containing 25 to 35 μg mL^−1^ silver induced a significant cytotoxicity already after 2 h incubation, indicating a faster silver nanoparticle dissolution in the presence of the platinum nanoparticles, in accordance with the dissolution results shown above.

Overall, the cell toxicity of the physical Ag/Pt 1 : 1 mixture at the same silver concentration was higher and occurred faster than for pure silver nanoparticles. This effect depended on incubation time and nanoparticle concentration. In a previous study we reported similar results regarding the antimicrobial activity of such Ag/Pt nanoparticle mixtures against *S. aureus* and *E. coli*. There, we found that the biological effects of the physical mixtures also depended on the ratio of silver and platinum nanoparticles. A significantly enhanced toxicity compared to pure silver nanoparticles was observed for alloyed nanoparticles with the compositions Ag/Pt 30 : 70 wt% and Ag/Pt 50 : 50 wt%. A platinum content of 30 wt% in the silver nanoparticles was insufficient to induce an enhanced toxicity. Dissolution experiments showed a fourfold increased silver ion release from physical mixtures of silver and platinum nanoparticles due to enhanced electrochemical activity.^22^ The oxidation of silver ions by dissolved oxygen in the absence of platinum is much slower and takes days to weeks.^14–16,19–21,29,43^

A likely explanation for the enhanced Ag^+^ release within the physical mixtures is a platinum-induced electrochemical sacrificial anode effect. The polarization is due to the different electronegativities of platinum (more noble) and silver (less noble). Furthermore, the exchange current density of platinum is much higher than that of silver, enhancing the reduction of oxygen at the platinum–water interface.^44^ For magnetron-sputtered Ag/Pt thin films at the micro- and nano-scale, this was demonstrated by Köller *et al.*^24^ and Abuayyash *et al.*^33^ A decrease in the size of silver nanoparticles within a physical mixture with platinum nanoparticles followed by the formation of bimetallic AgPt particles was reported by Hirakawa *et al.*^45^ In contrast, alloyed silver-platinum nanoparticles of about 15–25 nm diameter showed a release of silver only above 50 mol% silver content,^46^ confirmed by cell biological studies.^47^ This demonstrates that this electrochemical polarization effect in a physical mixture of nanoparticles cannot easily be transferred to alloyed nanoparticles where the metals are mixed on an atomic length scale. An increased cytotoxicity and microbial effect of ultrasmall alloyed silver-platinum nanoparticles (2 nm) was reported by Wolff *et al.*^48^ The fact that this effect was not accompanied by an increased dissolution (equivalent to a release of silver ions) suggested that ultrasmall nanoparticles exert their cytotoxic action not by the release of silver ions but inside a cell after uptake, probably due to their very small size.^48^

## Conclusions

Silver nanoparticles dissolve faster in the presence of platinum nanoparticles in aqueous electrolyte solution. The presence of both platinum nanoparticles and electrolyte ions in the dispersion medium is important as the immersion in pure water clearly shows. The dissolution occurs by direct contact of silver and platinum nanoparticles, leading to a positive polarization of the less-noble metal (silver) that enhances its dissolution (sacrificial anode effect). The presence of electrolyte ions in the immersion medium water leads to charge balancing, *i.e.* the electron transfer from silver to platinum. It is likely that the oxidizing species is dissolved oxygen that is reduced to hydroxide, taking up the electron from the silver.

## Conflicts of interest

There are no conflicts to declare.

## Data Availability

All data that were generated are included and shown in the article.

## References

[cit1] Eleraky N. E., Allam A., Hassan S. B., Omar M. M. (2020). Pharmaceutics.

[cit2] Liao C., Li Y., Tjong S. C. (2019). Int. J. Mol. Sci..

[cit3] Sabaghian H. (2024). ChemistrySelect.

[cit4] Aguilar-Garay R., Lara-Ortiz L. F., Campos-Lopez M., Gonzalez-Rodriguez D. E., Gamboa-Lugo M. M., Mendoza-Perez J. A., Anzueto-Rios A., Nicolas-Alvarez D. E. (2024). Pharmaceuticals.

[cit5] Roy S., Hasan I., Guo B. (2023). Coord. Chem. Rev..

[cit6] Wahab M. A., Li L. M., Matin M. A., Karim M. R., Aijaz M. O., Alharbi H. F., Abdala A., Haque R. (2021). Polymers.

[cit7] Abram S. L., Fromm K. M. (2020). Chem.–Eur. J..

[cit8] McGillicuddy E., Murray I., Kavanagh S., Morrison L., Fogarty A., Cormican M., Dockery P., Prendergast M., Rowan N., Morris D. (2017). Sci. Total Environ..

[cit9] Utembe W., Potgieter K., Stefaniak A. B., Gulumian M. (2015). Part. Fibre Toxicol..

[cit10] Schluesener J. K., Schluesener H. J. (2013). Arch. Toxicol..

[cit11] Chernousova S., Epple M. (2013). Angew. Chem., Int. Ed..

[cit12] Graf C., Nordmeyer D., Sengstock C., Ahlberg S., Diendorf J., Raabe J., Epple M., Köller M., Lademann J., Vogt A., Rancan F., Rühl E. (2018). Langmuir.

[cit13] Helmlinger J., Sengstock C., Gross-Heitfeld C., Mayer C., Schildhauer T. A., Köller M., Epple M. (2016). RSC Adv..

[cit14] Loza K., Epple M. (2018). RSC Adv..

[cit15] Adamczyk Z., Ocwieja M., Mrowiec H., Walas S., Lupa D. (2016). J. Colloid Interface Sci..

[cit16] Loza K., Diendorf J., Greulich C., Ruiz-Gonzales L., Gonzalez-Calbet J. M., Vallet-Regi M., Koeller M., Epple M. (2014). J. Mater. Chem. B.

[cit17] Batchelor-McAuley C., Tschulik K., Neumann C. C. M., Laborda E., Compton R. G. (2014). Int. J. Electrochem. Sci..

[cit18] Ahlberg S., Antonopulos A., Diendorf J., Dringen R., Epple M., Flöck R., Goedecke W., Graf C., Haberl N., Helmlinger J., Herzog F., Heuer F., Hirn S., Johannes C., Kittler S., Köller M., Korn K., Kreyling W. G., Krombach F., Lademann J., Loza K., Luther E. M., Malissek M., Meinke M. C., Nordmeyer D., Pailliart A., Raabe J., Rancan F., Rothen-Rutishauser B., Rühl E., Schleh C., Seibel A., Sengstock C., Treuel L., Vogt A., Weber K., Zellner R. (2014). Beilstein J. Nanotechnol..

[cit19] Liu J. Y., Wang Z. Y., Liu F. D., Kane A. B., Hurt R. H. (2012). ACS Nano.

[cit20] Kent R. D., Vikesland P. J. (2012). Environ. Sci. Technol..

[cit21] Zook J. M., Long S. E., Cleveland D., Geronimo C. L. A., MacCuspie R. I. (2011). Anal. Bioanal. Chem..

[cit22] Breisch M., Loza K., Pappert K., Rostek A., Rurainsky C., Tschulik K., Heggen M., Epple M., Tiller J. C., Schildhauer T. A., Köller M., Sengstock C. (2020). Nanotechnology.

[cit23] Abuayyash A., Ziegler N., Meyer H., Meischein M., Sengstock C., Moellenhoff J., Rurainsky C., Heggen M., Garzon-Manjon A., Scheu C., Tschulik K., Ludwig A., Köller M. (2020). Nanomed. Nanotechnol. Biol. Med..

[cit24] Koeller M., Bellova P., Javid S. M., Motemani Y., Khare C., Sengstock C., Tschulik K., Schildhauer T. A., Ludwig A. (2017). Mater. Sci. Eng., C.

[cit25] Veronesi G., Deniaud A., Gallon T., Jouneau P. H., Villanova J., Delangle P., Carrière M., Kieffer I., Charbonnier P., Mintz E., Michaud-Soret I. (2016). Nanoscale.

[cit26] Ho C. M., Wong C. K., Yau S. K. W., Lok C. N., Che C. M. (2011). Chem.–Asian J..

[cit27] Liu L., Hurt R. H. (2010). Environ. Sci. Technol..

[cit28] Liu J., Sonshine D. A., Shervani S., Hurt R. H. (2010). ACS Nano.

[cit29] Kittler S., Greulich C., Diendorf J., Köller M., Epple M. (2010). Chem. Mater..

[cit30] Ho C. M., Yau S. K. W., Lok C. N., So M. H., Che C. M. (2010). Chem.–Asian J..

[cit31] Stein S., Kruck L., Warnecke D., Seitz A., Dürselen L., Ignatius A. (2020). Eur. Cells Mater..

[cit32] Chen L., Song X. Y., Xing F., Wang Y. A., Wang Y. Z., He Z. Y., Sun L. (2020). J. Biomed. Nanotechnol..

[cit33] Abuayyash A., Ziegler N., Gessmann J., Sengstock C., Schildhauer T. A., Ludwig A., Köller M. (2018). Adv. Eng. Mater..

[cit34] Rostek A., Breisch M., Pappert K., Loza K., Heggen M., Köller M., Sengstock C., Epple M. (2018). Beilstein J. Nanotechnol..

[cit35] Thust A., Barthel J., Tillmann K. (2016). Journal of Large-Scale Research Facilities.

[cit36] Heggen M., Luysberg M., Tillmann K. (2016). Journal of Large-Scale Research Facilities.

[cit37] Chantler C. T., Tran C. Q., Cookson D. J. (2004). Phys. Rev. A: At., Mol., Opt. Phys..

[cit38] Klug H. P. and Alexander L. E., X-ray diffraction procedures for polycrystalline and amorphous materials, Wiley-Interscience, New York, 1974

[cit39] Yu R., Liz-Marzan L. M., Garcia de Abajo F. J. (2017). Chem. Soc. Rev..

[cit40] Settem M., Islam M., Kanjarla A. K. (2018). Comput. Mater. Sci..

[cit41] Loza K., Sengstock C., Chernousova S., Koeller M., Epple M. (2014). RSC Adv..

[cit42] Xiu Z. M., Zhang Q. B., Puppala H. L., Colvin V. L., Alvarez P. J. J. (2012). Nano Lett..

[cit43] Dong F., Valsami-Jones E., Kreft J. U. (2016). J. Nanopart. Res..

[cit44] Uhlig H. H. , Corrosion and Corrosion Control, Wiley, New York, London, 1963

[cit45] Hirakawa K., Kaneko T., Toshima N. (2018). Chem.–Asian J..

[cit46] Grasmik V., Breisch M., Loza K., Heggen M., Köller M., Sengstock C., Epple M. (2018). RSC Adv..

[cit47] Breisch M., Grasmik V., Loza K., Pappert K., Rostek A., Ziegler N., Ludwig A., Heggen M., Epple M., Tiller J. C., Schildhauer T. A., Koller M., Sengstock C. (2019). Nanotechnology.

[cit48] Wolff N., Bialas N., Loza K., Heggen M., Schaller T., Niemeyer F., Weidenthaler C., Beuck C., Bayer P., Prymak O., Oliveira C. L. P., Epple M. (2024). Materials.

